# Platelet Satellitism

**DOI:** 10.4274/tjh.galenos.2019.2019.0171

**Published:** 2020-02-20

**Authors:** Yasemin Ardıçoğlu Akışın, Nejat Akar

**Affiliations:** 1 TOBB-ETU Faculty of Medicine, Department of Biochemistry, Ankara, Turkey; 2 TOBB-ETU Faculty of Medicine, Department of Pediatrics, Ankara, Turkey

**Keywords:** Platelet, Polymorphonuclear leukocytes, Satellitism

Platelet satellitism is a rare in vitro phenomenon that occurs when an immunoglobulinG antibody directed against the glycoprotein IIb/IIIa complex on the platelet membrane forms in ethylenediaminetetraacetic acid-treated peripheral blood at room temperature [1,2]. As the antibody coats the platelets, platelets adhering to polymorphonuclear leukocytes show a rosette-like appearance [3]. There is no definite causal association with any disease. Severe rosetting may lead to a misdiagnosis of thrombocytopenia unless peripheral smears are examined [4].

The images presented here were obtained from a 5-year-old child with an upper respiratory tract infection accompanying asthma. Thrombocytopenia was not detected in complete blood count (Figures 1 and 2).

## Figures and Tables

**Figures 1 and 2:**
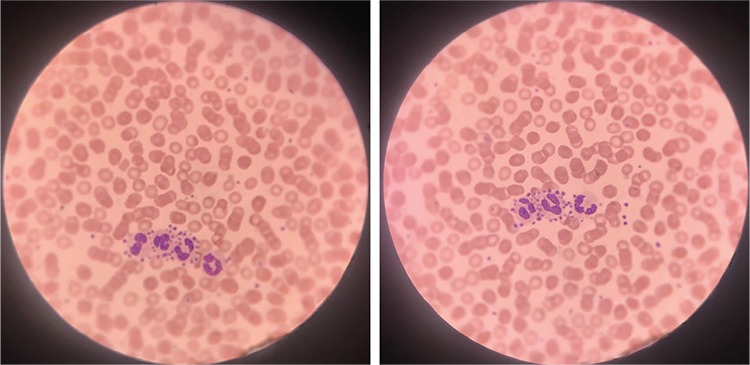
Platelet satellitism in a 5-year-old child with an upper respiratory tract infection accompanying asthma.
